# Evaluation of a Yeast–Polypyrrole Biocomposite Used in Microbial Fuel Cells

**DOI:** 10.3390/s22010327

**Published:** 2022-01-02

**Authors:** Antanas Zinovicius, Juste Rozene, Timas Merkelis, Ingrida Bruzaite, Arunas Ramanavicius, Inga Morkvenaite-Vilkonciene

**Affiliations:** 1 Department of Mechatronics, Robotics and Digital Manufacturing, Vilnius Gediminas Technical University, 03224 Vilnius, Lithuania; antanas.zinovicius@vilniustech.lt (A.Z.); juste.rozene@vilniustech.lt (J.R.); timas.merkelis@chgf.stud.vu.lt (T.M.); 2 Department of Chemistry and Bioengineering, Faculty of Fundamental Sciences, Vilnius Gediminas Technical University, 10223 Vilnius, Lithuania; ingrida.bruzaite@vilniustech.lt; 3 Department of Physical Chemistry, Faculty of Chemistry and Geosciences, Vilnius University, 03225 Vilnius, Lithuania; 4 Laboratory of Nanotechnology, State Research Institute Centre for Physical Sciences and Technology, 02300 Vilnius, Lithuania; 5 Laboratory of Electrochemical Energy Conversion, State Research Institute Centre for Physical Sciences and Technology, 10257 Vilnius, Lithuania

**Keywords:** polypyrrole, conducting polymers, biofuel cells, yeast, cell modification, cell wall, cell membrane, atomic force microscopy (AFM), cell morphology, cyclic voltammetry

## Abstract

Electrically conductive polymers are promising materials for charge transfer from living cells to the anodes of electrochemical biosensors and biofuel cells. The modification of living cells by polypyrrole (PPy) causes shortened cell lifespan, burdens the replication process, and diminishes renewability in the long term. In this paper, the viability and morphology non-modified, inactivated, and PPy-modified yeasts were evaluated. The results displayed a reduction in cell size, an incremental increase in roughness parameters, and the formation of small structural clusters of polymers on the yeast cells with the increase in the pyrrole concentration used for modification. Yeast modified with the lowest pyrrole concentration showed minimal change; thus, a microbial fuel cell (MFC) was designed using yeast modified by a solution containing 0.05 M pyrrole and compared with the characteristics of an MFC based on non-modified yeast. The maximal generated power of the modified system was 47.12 mW/m^2^, which is 8.32 mW/m^2^ higher than that of the system based on non-modified yeast. The open-circuit potentials of the non-modified and PPy-modified yeast-based cells were 335 mV and 390 mV, respectively. Even though applying a PPy layer to yeast increases the charge-transfer efficiency towards the electrode, the damage done to the cells due to modification with a higher concentration of PPy diminishes the amount of charge transferred, as the current density drops by 846 μA/cm^2^. This decrease suggests that modification by PPy may have a cytotoxic effect that greatly hinders the metabolic activity of yeast.

## 1. Introduction

There is a very close link between electrochemical sensor technologies and those used to develop biofuel cells [1,2,3]. Microbial fuel cells (MFCs) convert chemical energy into electric power via microorganism-catalysed reactions [4,5]. Among many other applications, MFCs are promising for treating wastewater and waste from the food industry. The attainable metabolic energy from such devices can partially meet ever-rising energy demands by reducing industry-generated waste in cheap, clean, and renewable ways, without producing toxic by-products. The electric charge from the ongoing substrate oxidation process during microorganisms’ metabolic processes is released and then transferred towards the electrodes [5]. Microbial fuel cells require continuous electric charge release in the anode and consumption at the cathode in order to generate electrical power [6]. The MFC efficiency greatly depends on the flow of electric charge transfer from the microorganisms to the electrodes [7,8]. Many new materials applied for electrode modification are used to enhance cell performance by increasing electric charge transfer [5,9,10,11,12].

The yeast *Saccharomyces cerevisiae* is widely used in microbial fuel cell construction as a biochemical reaction catalyst [13,14,15,16]. This microorganism, also known as baker’s yeast, is commonly used in the food industry; as a result, it appears suitable for use with MFCs in industrial waste treatment [14]. Moreover, yeast cells are extensively used as a model organism to model the performance of eukaryotic cells. They are widely investigated, have a fast reproduction rate and high metabolic efficiency, are inexpensive and non-pathogenic, and can be cultivated under simple growth conditions [17,18]. *S. cerevisiae* has different membranes: (1) a plasma membrane, by which the cell is separated from the environment; and (2) a transmembrane electron transfer protein and mitochondrial membrane, which contains proteins connected with metabolism [19]. Yeast uses mono- and disaccharides as carbon sources, which are oxidised by forming two final products–CO_2_, and H_2_O—which are both generated over numerous intracellular redox reactions. Bioenergy is usually transferable via redox reactions induced by other materials with high redox activity, e.g., redox mediators. Due to the complex yeast cell structure, a double-mediator system should be used [20,21]. One of these redox mediators is lipophilic, which can cross all cell membranes and join intracellular redox processes; the second is the hydrophilic redox mediator, which transfers electric charge from the extracellular environment towards the electrode. In such a system, the bioenergy can be converted to usable electrical power. However, in practice, these MFCs generate low amounts of electrical power. One of the possible solutions is to increase charge-transfer efficiency while modifying microorganisms with conducting polymers, which preserve the viability of living cells [22,23]. Redox-active polymers such as polypyrrole (PPy) are promising materials for mediating charge transfer compared to carbon nanotubes or modified organic matrices, due to their chemical versatility, tuneable conductivity, reversible electrochemical processes, controllable nanostructure, and low-cost synthesis [24,25,26]. PPy is a biocompatible material suitable for surface modification of cells and nanoparticles, assisting in drug delivery, and enhancing deep-tissue imaging [27]. This offers a significant advantage over high-cost carbon nanotubes and modified organic matrices with lower capacitance and redox activity [28]. Approaches using redox-active polymers are attracting more and more attention due to their being rather efficient, providing biosensor stability and reagent-free assay with no mediator wash-out during the procedure, and being usable for creating biosensors based on microbial cells [29]. Additionally, carbon nanotubes and conductive polymers can be used together via covalent functionalisation to customise their properties [30]. However, the effect of PPy on cells is as-yet unknown: it could increase cell wall conductivity, permeability, or both. In our previous research, the periplasm of *Aspergillus niger* was modified by encapsulating it with PPy, and the results showed a noticeable increase in current values [17,31]. Furthermore, in yeast-based MFCs, the most commonly used hydrophilic mediator is [Fe(CN)_6_]^3−^/[Fe(CN)_6_]^4−^. Low amounts of [Fe(CN)_6_]^3−^ are redox-cycled in the yeast plasma membrane as it approaches the transmembrane electron transfer protein, and this cycling can initiate pyrrole polymerisation in between the cell wall and the plasma membrane, causing self-modification [32]. This modification forms a yeast–PPy biocomposite, which can be further used in MFCs, increasing their effectiveness. However, it is unclear how these redox mediators affect cell viability, physiological state, and metabolic activity.

Cell size and morphology can be considered when evaluating yeast cells’ physiological state (e.g., form irregularities, diameter, and roughness) [33,34]. Typically, lower viability causes a decrease in cell diameter and increases the roughness of the surface. Encapsulation of yeast cells in PPy could potentially disrupt the cell wall and cause a change in the cell’s topology and morphology [35]. PPy-modified yeast cells are more resilient to sudden environmental changes, since their stiffness significantly increases [36].

The main aim of this research was to evaluate the effectiveness of microbial fuel cells using a yeast–PPy biocomposite. Studies of modified and non-modified yeast’s electrochemical activity, along with the topology and morphology of the cells modified with different PPy concentrations, were carried out to achieve this goal.

## 2. Materials and Methods

### 2.1. Materials

Potassium ferricyanide and potassium ferrocyanide (>99%) were purchased from Carl Roth; hydrogen peroxide (30%) was purchased from Riedel; sulfuric acid (98%) was purchased from Alfa Aesar; 9,10-phenantrenequinone, YPD broth, pyrrole (98%), D-(+)-glucose (99%), and phosphate-buffered saline (PBS) tablets were purchased from Merck; and ethanol (98%) was purchased from Spiritus Vilnensis. Dry *Saccharomyces cerevisiae* yeast was purchased from the food supplier “Dr. Oetker Lietuva”.

Briefly, 0.1 M PBS buffer was prepared by dissolving the tablet in distilled water (pH 6.8), while 0.4 M potassium ferricyanide, 0.4 M potassium ferrocyanide, and 1 M glucose were prepared in a buffer solution. Glucose solution was left for 24 h before use for glucose molecule mutarotation. The 4 mM 9,10-phenantrenequinone (PQ) solution was prepared in ethanol.

### 2.2. Yeast Preparation and Modification

YPD broth was mixed with PBS to obtain a 50 g/L concentration of YPD medium. Next, dry *Saccharomyces cerevisiae* yeast was added to the prepared medium for a 10 mg/mL concentration. The medium with yeast was incubated at 32 °C on an orbital shaker at 250 rpm for 17–24 h (until the yeast reached the logarithmic phase). Yeast was then harvested by centrifugation at 5600 rpm for 3 min and washed with PBS 3 times. The wet mass was weighed and suspended in PBS to a concentration of 0.5 g/mL.

The suspension of cells was placed in an ultrasound bath (Bandelin Sonorex RK100, Berlin, Germany) for 5 min to inactivate the yeast cells.

For modification of *Saccharomyces cerevisiae* with PPy, the solution containing 0.3 M pyrrole, 0.4 M potassium ferrocyanide, 1 M glucose, and PBS was used and incubated at 32 °C on an orbital shaker at 250 rpm for 24 h. The cell harvesting and suspension preparation parameters and conditions were the same as those applied for yeast preparation.

### 2.3. Yeast Cell Viability Assays

Analysis of the efficiency of pyrrole for yeast cells proliferation: *Saccharomyces cerevisiae* yeast cells grown overnight (1 mL) were mixed with YPD medium (8 mL) and cultivated for 4 h at 32 °C (optical density (OD) was observed at 600 nm, and was equal to 0.49). Then, 100 µL of 0.3 M pyrrole solution was added to the prepared yeast cell suspensions and incubated for 24 h under shaking at 120 rpm and 32 °C; in the control sample, pyrrole was absent. The OD of the yeast cell cultures was observed for 24 h, with 1 h intervals between measurements. The number of yeast cells was monitored on liquid YPD media by measuring the light absorbance using a GENESYS 10S UV–Vis spectrophotometer from Thermo Fisher Scientific (Mettler Toledo, Singapore).

Serial dilutions were performed in 0.9% NaCl, and 50 µL of each solution was spread onto YPD agar plates, followed by overnight incubation at 32 °C and counting of colony-forming units (CFUs).

### 2.4. Atomic-Force-Microscopy-Based Measurements

A silicon wafer with hollow wells (squares with borders ranging from 5 to 10 µm) was immersed in acidic piranha solution (H_2_SO_4_ 3:1 H_2_O_2_) for 15 min; it was then washed with a large volume of distilled water and dried. Finally, 0.5 µL of yeast cell solution was dropped on the wafer and left until it was visually dry.

The BioScope II AFM combined with an inverted optical microscope developed by Veeco Instruments Ltd. (Santa Barbara, CA, USA) and an NP-D cantilever (Bruker, Camarillo, CA, USA) was used for the measurements. AFM contact mode in the phosphate buffer solution was applied at 0.15 Hz. Experiments were performed with the PPy-modified, non-modified, and inactivated yeast cells. Raw data were analysed using NanoScope Analysis 1.5 software.

### 2.5. Electrochemical Measurements

Yeast Cell Immobilisation: A graphite electrode (rod with 150 mm length, 3 mm diameter, low density, 99.995% trace metals basis) was cut into 30 mm long pieces. The ends were sanded down with different grid sizes of sandpaper and polished with paper until a shining surface was achieved, after which they were washed with ethanol and water to remove any remaining residues. Next, 2.5 μL of 9,10-phenantrenequinone solution was deposited onto the shiny electrode surface. After the PQ dried, a 2.5 μL drop of the non-modified or PPy-modified yeast suspension was placed onto the electrode. The modified electrode was covered by a polycarbonate membrane with a pore size of 3 µm. Electrodes prepared via this procedure were used for both electrochemical measurements and the microbial fuel cell anode. The microbial fuel cell cathode was based on bare graphite with a much higher surface area than the anode.

Electrochemical measurements were performed using a Metrohm μStat-i 400 Potentiostat/Galvanostat (Utrecht, The Netherlands) and DropView software. All experiments were carried out at ambient temperature (20 °C). The electrochemical cell consisted of three electrodes: a graphite electrode was connected as the working electrode, a platinum electrode as the counter electrode, and a Ag/AgCl (KCl, 3 M) electrode as the reference electrode. All of the components (borosilicate glass titration vessel with a plastic mounting ring and lid, platinum, and 12.5 cm long Ag/AgCl (KCl, 3 M) electrodes) were purchased from Metrohm AG (Herisau, Switzerland). If the concentration of the glucose or other materials was changed during the measurements, it was done sequentially by adding it to the same electrochemical cell.

A single potential pulse of 1 V vs. Ag/AgCl lasting for 10 s was applied in the electrochemical cell with the 50 mM pyrrole solution for electrochemical formation of PPy.

Four cyclic voltammetry cycles were carried out for each experiment with a rate of potential from −0.6 V to 0.6 V, a scan rate of 50 mV/s, and a step size of 10 mV. Only the final cyclic voltammetry cycles were plotted.

Microbial fuel cell experiments were conducted in 0.1 M phosphate buffer, pH 6.8, containing 30 mM glucose under aerobic conditions. Measurements were performed in the two-electrode electrochemical cell with a differently modified graphite electrode as the anode, and a graphite electrode with several times greater working area as the cathode. Voltage measurements in the double-compartment-designed MFC were performed with external resistances (of 0.01 kΩ 0.1 kΩ; 0.42 kΩ; 0.9 kΩ; 5 kΩ; 12 kΩ; 50 kΩ; 100 kΩ; 400 kΩ; 800 kΩ; 1100 kΩ; 1500 kΩ; 2100 kΩ; and 2450 kΩ) to imitate external load on the cell and accurately assess power density.

### 2.6. Calculations

Roughness can be described by height, wavelength, spacing, and hybrid parameters [37]; among these, the most significant parameter is height, which consists of many elements [38]:

$R_{a}$ is the arithmetic average [39]:

(1) $$R_{a}=\frac{1}{L}\int_{0}^{L}\left|Z\left(x\right)\right|dx$$

$R_{q}$ is the root-mean-square roughness [40]:

(2) $$R_{q}=\sqrt{\frac{1}{L}\int_{0}^{L}\left|Z^{2}\left(x\right)\right|dx}$$

$R_{p}$ is the maximum profile peak height [40]:

(3) $$R_{p}=\left|maxZ\left(x\right)\right|,$$

$R_{v}$ is the minimum profile valley depth [40]:

(4) $$R_{v}=\left|minZ\left(x\right)\right|,$$

$R_{T}$ is the maximum height of the profile [40]:

(5) $$R_{T}=R_{p}+R_{v},$$

where Z(x) is the function describing the surface profile in terms of height (Z) and position (x) over the evaluated length (L).

Electrochemical measurements were evaluated by applying Hill’s function:

(6) $$J=\frac{C^{n}}{k^{n}+C^{n}}$$

where J is the current density, C is the glucose concentration, k is a constant equal to the concentration when half of the maximal current is observed, and n is Hill’s coefficient describing species cooperativity.

## 3. Results and Discussion

### 3.1. Evaluation of Yeast Cell Viability

**Figure 1:**
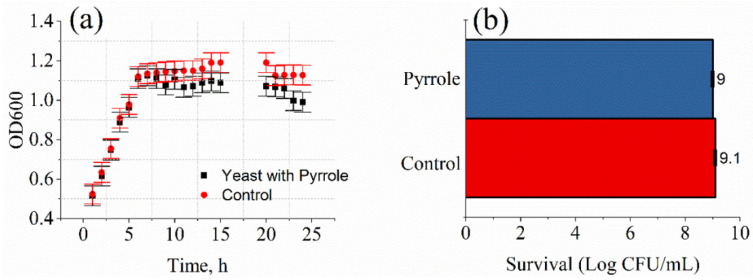
The viability of *Saccharomyces cerevisiae* yeast cells under the action of 0.3 M pyrrole solution: (**a**) Growth curves of *Saccharomyces cerevisiae* yeast cells:Control—yeast cells without pyrrole; yeast cells with 0.3 M pyrrole. (**b**) Effect of pyrrole solution on the formation of yeast cell colonies. The data are given as log CFU/mL survival.

The possible effect of 0.3 M pyrrole solution on the yeast cells was determined spectroscopically by the evaluation of the OD of the cells’ suspension. During the studies on the mode of antifungal action of the formed PPy, we found that the growth curve of *Saccharomyces cerevisiae* yeast cells changed slightly. Optical density measurements of *Saccharomyces cerevisiae* yeast cells grown in the liquid YPD medium with or without pyrrole solution are presented in Figure 1.

After 8 h of cultivating yeast cells, optical density was lower in *Saccharomyces cerevisiae* aliquots containing 0.3 M pyrrole solution (1.11 ± 0.05). and continued to decline compared to the control solution of yeast cells (1.14 ± 0.05). The antimicrobial activity of pyrrole solution was investigated against both control yeast and yeast modified with polypyrrole, using the counting of colony-forming units (CFUs) (Figure 1b). Following 24 h incubation of test samples, only ~1 log CFU/mL reduction in yeast cells was recorded. Several studies on the antifungal potential of polypyrrole suggest that results vary significantly, from low to high [20].

### 3.2. Evaluation of Yeast Cells’ Morphological Properties and Surface

**Figure 2:**
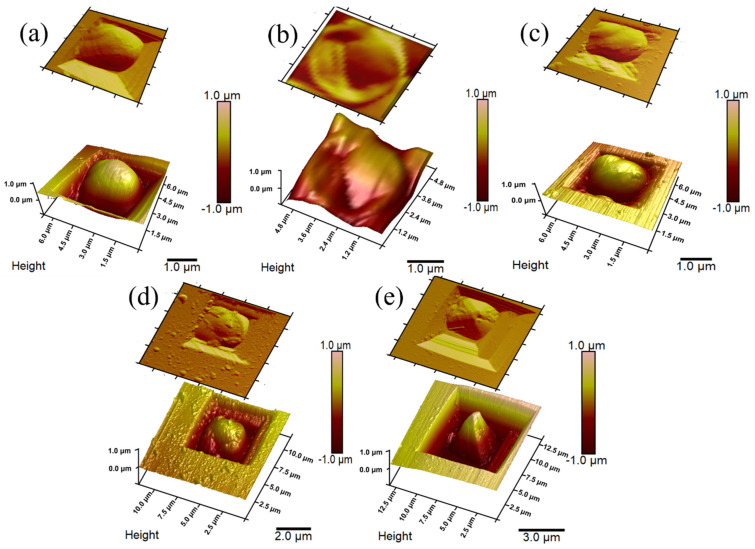
AFM images: (**a**) Non-modified yeast cell; (**b**) inactivated yeast cell; (**c**) PPy_0.05_-modified yeast cell; (**d**) PPy_0.1_-modified yeast cell; (**e**) PPy_0.3_-modified yeast cell.

Three types of single yeast cells—non-modified (Figure 2a), inactivated (Figure 2b), and PPy-modified yeast cells with different pyrrole concentrations (Figure 2c–e)—immobilised in silicon hollows, were scanned by AFM. The topography of yeast cells modified by a solution containing 0.05 M or 0.1 M pyrrole (Figure 2c,d, respectively) showed small agglomerations, which were abnormal compared to non-modified (Figure 2a) and inactivated (Figure 2b) cells. Polymer clusters become more significant with increased PPy concentration. These changes can be explained as an effect of PPy modification. However, no agglomerations were observed at the highest tested concentration (0.3 M PPy), most likely because the polypyrrole formed a uniform layer; only the shrinkage of the cell was observed (Figure 2e).

**Figure 3:**
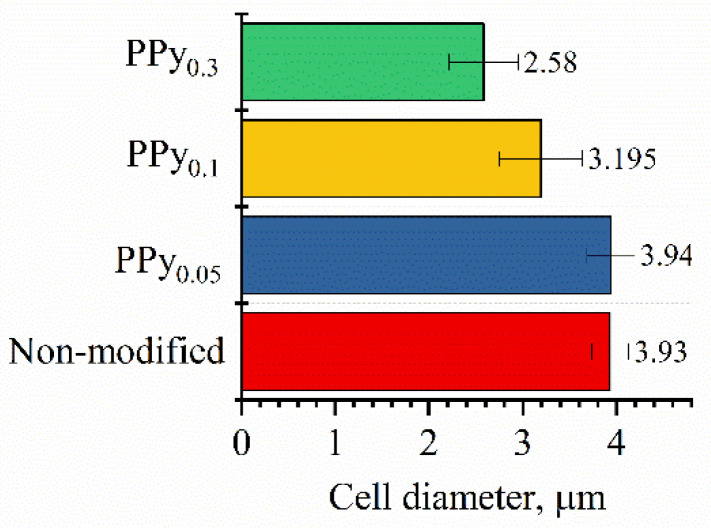
Dependence of cell diameter on the concentration of polypyrrole.

According to the cell size change results (Figure 3), the increasing PPy concentration at the modification step significantly affected the cell; cells became smaller (diameter from 3.93 µm to 2.58 µm), leading to some restrictions of the budding process. Non-modified yeast cells were ~4 µm in diameter, with low deviation, while after the increase in PPy concentration, cells became smaller, with increased deviations in cell diameter.

**Figure 4:**
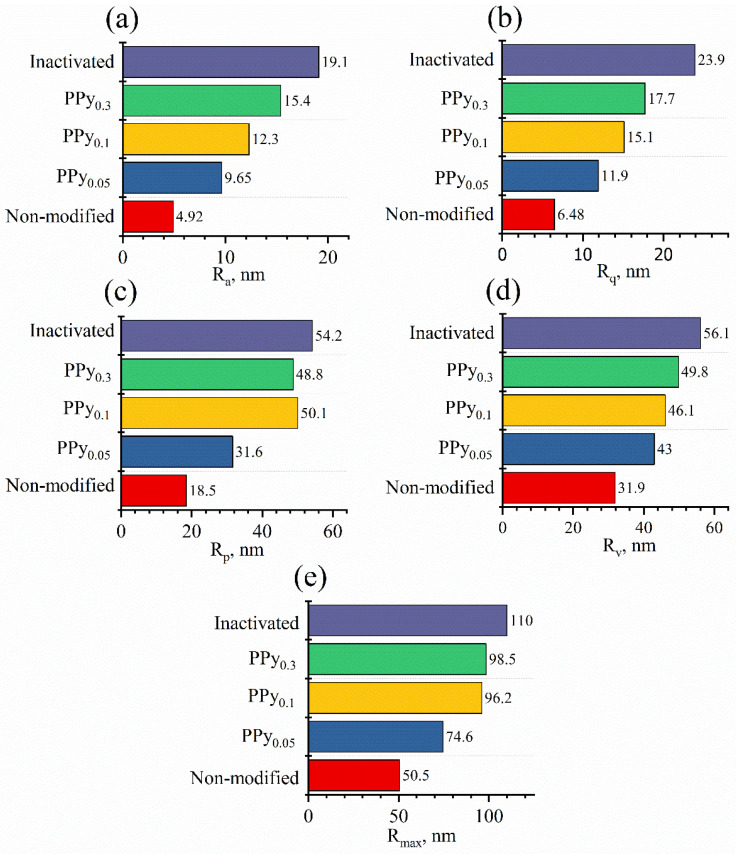
Surface roughness parameters’ dependence on PPy concentration: (**a**) *R_a_*—arithmetic average; (**b**) *R_q_*—root-mean-square roughness; (**c**) *R_p_*—maximum height of the peaks; (**d**) *R_v_*—maximum depth of the valleys; (**e**) *R_max_*—maximum height of the profile.

After assessing yeast cell size, 500 × 500 nm topographical images were taken, and cell wall roughness was investigated. The results show an increase in roughness parameters with the increase in the pyrrole concentration used for the modification (Figure 4). Comparing modified cells shows that changing the pyrrole concentration in the solution used for yeast cell modification from 0.05 M to 0.3 M yielded 1.6 times greater roughness when comparing $R_{a}$, 1.5 times greater roughness when comparing $R_{q}$ and $R_{p}$, 1.2 times greater roughness when comparing $R_{v}$, and 1.3 times greater roughness when comparing $R_{max}$.

When increasing the PPy concentration, all roughness parameters approached the roughness of inactivated yeast cells. Modifying yeast cells with high PPy concentrations might decrease cell longevity and slowly inactivate them.

### 3.3. Evaluation of Yeast Cells’ Metabolic Activity by Cyclic Voltammetry

**Figure 5:**
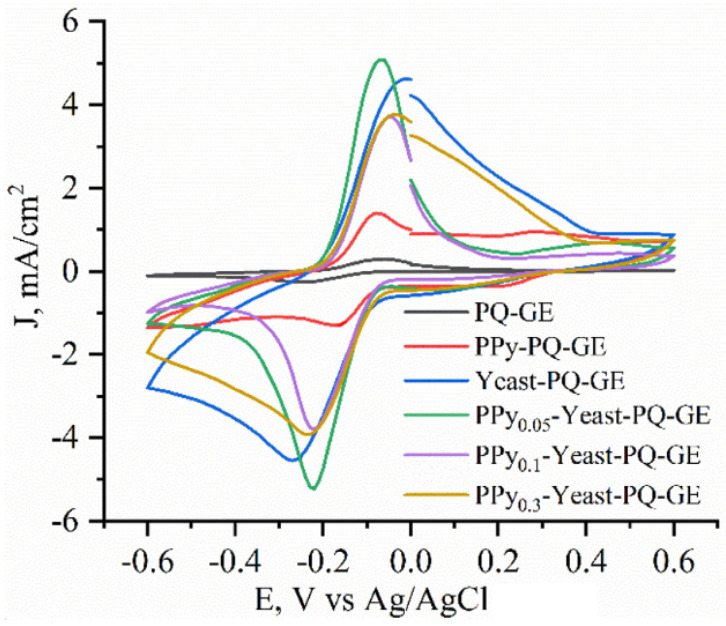
Cyclic voltammograms of a bare graphite electrode (GE) modified with PQ, a PPy–PQ-modified GE, a yeast–PQ-modified GE, and a PPy–yeast–PQ-modified GE at different pyrrole concentrations. Measurements were performed in phosphate buffer solution with 0.75 mM potassium ferricyanide and 50 mM glucose.

Further experiments investigated the effect on metabolic activity when yeast cells were modified with different PPy concentrations. The cyclic voltammograms of non-modified, various concentrations of PPy-modified, bare, and pure-PPy-modified graphite electrodes were recorded (Figure 5) when glucose, PQ, and potassium ferricyanide concentrations remained constant.

Current density increased by 455 μA/cm^2^ when comparing non-modified yeast to yeast modified with a solution containing 0.05 M PPy, thus hinting that PPy can increase charge-transfer efficiency. However, when the PPy concentration used for yeast modification was further increased to 0.1 M, the current density dropped by 846 μA/cm^2^, while a further increase to 0.3 M produced a minimal change in current density, suggesting that modification may have cytotoxic effects, hindering cells’ activity and slowing down their metabolic activity.

**Figure 6:**
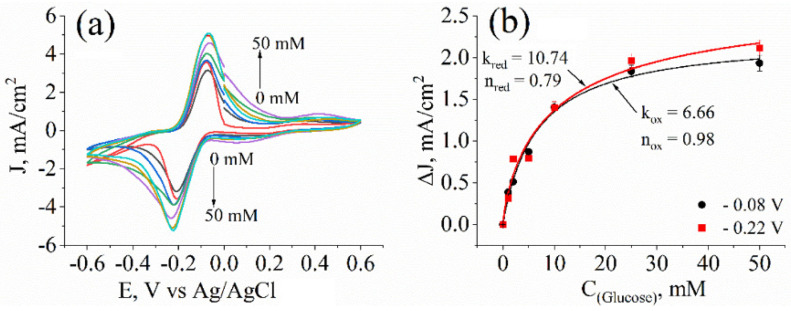
(**a**) Cyclic voltammograms of PPy−yeast−PQ-modified graphite electrodes at different glucose concentrations (from 0 to 50 mM). Measurements were performed in a buffer solution with 0.75 mM potassium ferricyanide. (**b**) Current density dependencies on glucose concentration calculated from the peak current at (**a**). Lines represent mathematical-modelling-based results generated using Hill’s function (Equation (6)), which are fitted to experimental data. Measurements were performed in a three-electrode electrochemical cell.

For further experiments, modification of yeast with the solution containing 0.05 M PPy was chosen to evaluate cell metabolic activity at different glucose concentrations when PQ and potassium ferricyanide concentrations were kept constant. In addition, the steady-state cyclic voltammograms were evaluated (Figure 6a). When glucose concentration increased from 0 to 50 mM, the current density reached the maximum value of 5.2 mA/cm^2^ at 25 mM glucose. When glucose concentration was increased beyond this level, the current density change was marginal.

The peaks of the oxidation (at −0.08 V) and reduction (at −0.22 V) changes in current density from glucose-dependent cyclic voltammograms were plotted as a concentration–dependence curve and evaluated by fitting to Hill’s function (Equation (6) and Figure 6b) to determine the dependency of current density on glucose concentration. The Hill coefficient “n” when considering a reduction in both cases was lower than 1 (n_red_ = 0.79, n_ox_ = 0.98), indicating negative cooperativity [21]. This could hint that the PPy encapsulation impaired the entry of glucose into the cell. As Hill coefficient “n” for the oxidation process approaches 1, negative cooperativity is negligible. The constant “k”, which shows the concentration at which half of the maximal current is registered, was lower at the oxidation (k_ox_ = 6.66) than at the reduction potential (k_red_ =10.74), which indicates that the oxidation reaction rate was faster.

### 3.4. Assessment of Microbial Fuel Cell Performance

**Figure 7:**
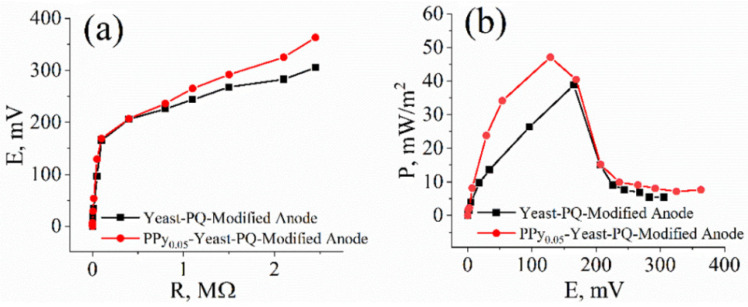
(**a**) Dependence of potential on the applied external load. (**b**) Calculated dependence of power density on the generated potential in the double-compartment-based MFC, which consisted of (1) a PPy_0.05_–yeast–PQ-modified anode and (2) a bare graphite cathode immersed in the phosphate buffer solution containing 0.75 mM potassium ferricyanide and 30 mM glucose.

To evaluate PPy-modified yeast as a potential catalyst in MFCs, a microbial fuel cell was constructed based on a two-electrode electrochemical system, where the anode is graphite modified with PQ and solution containing 0.05 M PPy, or non-modified yeast cells, and the cathode is a bare graphite electrode with much higher surface area compared to that of the anode. MFC performance results (Figure 7) were consistent with electrochemical analysis data. Yeast modified with PPy (47.12 mW/m^2^) generated higher maximum power output than non-modified (38.8 mW/m^2^) yeast cells, with a difference of 8.32 mW/m^2^. The open-circuit potentials of the non-modified and PPy-modified yeast-based cells were 335 mV and 390 mV, respectively.

To increase the charge-transfer efficiency, the electrode surface was modified with poly(neutral red) polymer on a carbon felt surface when the yeast was in solution (not immobilized), and a maximal power output of 6.1 mW/m^2^ was achieved [41]. Polypyrrole nanoparticles were synthetized on the graphite felt electrode and used for the enhancement of charge transfer from *Shewanella putrefaciens* towards the electrode. Haribabu et al. achieved a maximal power output of 1.22 W/m^2^ [42]. The maximal calculated power output of the PPy_0.05_-modified yeast-based cell was 47.12 mW/m^2^ at 129 mV (Figure 7b). The results show that modification with a solution containing 0.05 M pyrrole increases the charge-transfer efficiency between the yeast cells and the electrode. Thus, pyrrole has the potential to be applied in the further development of microbial fuel cells.

## 4. Conclusions

Modification of yeast with polypyrrole (PPy) is a promising way to increase the charge transfer from yeast to the electrode. However, the modification with PPy burdens the cell replication process to a certain degree, causing the cells to become smaller. From AFM images obtained from the cells modified with different concentrations of PPy, it can be seen that when the concentration of pyrrole in the solution used for the modification of yeast increases, the cell size declines. In addition, polymer clusters, which are visible on cells’ topography, appear after the modification. However, from the AFM images, it is not clear where they are formed—in the periplasm, or on the top of the cell wall.

Furthermore, after modifying the cell with different PPy concentrations, an increase in roughness was observed, and surface roughness parameters at the 0.1 M PPy concentration became closer to those of the inactivated cells. Our results show that cell modification with a solution containing 0.05 M pyrrole causes minor changes in cells’ size, shape, form, and surface roughness parameters, making this concentration the most suitable for further use for yeast modification in biofuel cell anodes. The electrochemical analysis also shows that only the solution applied for the modification containing 0.05 M pyrrole increased the charge-transfer efficiency. This result suggests that the modification with solutions containing 0.1 M and 0.3 M pyrrole may have cytotoxic effects, hindering cell activity and slowing down metabolic activity or obstructing glucose supply. Therefore, the microbial fuel cell was designed with an anode modified with PQ and yeast cells modified with a solution containing 0.05 M PPy. The maximal calculated power output of the designed cell was 47.12 mW/m^2^ at 129 mV. This outcome shows that modification of yeast cells with low concentrations of PPy is a viable option when developing a microbial fuel cell.
